# Supplementary material for: Quality, Equity and Utility of Observational Studies during 10 Years of Implementing the Structured Operational Research and Training Initiative in 72 Countries
Source: Trop Med Infect Dis. 2020 Nov 6;5(4):167. doi: 10.3390/tropicalmed5040167 (PMC7709644; doi:10.3390/tropicalmed5040167)
Supplement: Supplementary file 1 [file tropicalmed-05-00167-s001.pdf]

Supplementary material of Quality, Equity and Utility of Observational Studies during 10 Years of Implementing the Structured Operational Research and Training Initiative in 72 Countries

Table S1. STROBE Statement—adapted checklist of items to be included in reports of observational studies. Items 23 and 24 were added by TDR.

	Item No.	Recommendation	Reported on Page No.
**Title and abstract**	1	(*a*) Indicate the study's design with a commonly used term in the title or the abstract	
		(*b*) Provide in the abstract an informative and balanced summary of what was done and what was found	
		**Introduction**	
Background/rationale	2	Explain the scientific background and rationale for the investigation being reported	
Objectives	3	State specific objectives, including any prespecified hypotheses	
		**Methods**	
Study design	4	Present key elements of study design early in the paper	
Setting	5	Describe the setting, locations, and relevant dates, including periods of recruitment, exposure, follow-up, and data collection	
Participants	6	(*a*) *Cohort study*—Give the eligibility criteria, and the sources and methods of selection of participants. If applicable, describe methods of follow-up<br>*Case-control study*—Give the eligibility criteria, and the sources and methods of case ascertainment and control selection. Give the rationale for the choice of cases and controls<br>*Cross-sectional study*—Give the eligibility criteria, and the sources and methods of selection of participants	
		If applicable;<br>(*b*) *Cohort study*—For matched studies, give matching criteria and number of exposed and unexposed<br>*Case-control study*—For matched studies, give matching criteria and the number of controls per case	
Variables	7	Clearly define all outcomes, exposures, predictors, potential confounders, and effect modifiers. Give diagnostic criteria, if applicable	
Data sources/ measurement	8*	For each variable of interest, give sources of data and details of methods of assessment	

		(measurement). Describe comparability of assessment methods if there is more than one group
Bias	9	Describe any efforts to address potential sources of bias
Study size	10	Explain how the study size was arrived at

Quantitative variables	11	Explain how quantitative variables were handled in the analyses. If applicable, describe which groupings were chosen and why
Statistical methods	12	(*a*) Describe all statistical methods, and if applicable those used to control for confounding
		(*b*) If applicable, describe any methods used to examine subgroups and interactions
		(*c*) If applicable, explain how missing data were addressed.
		(*d*) *Cohort study*—If applicable, explain how loss to follow-up was addressed<br>*Case-control study*—If applicable, explain how matching of cases and controls was addressed<br>*Cross-sectional study*—If applicable, describe analytical methods taking account of sampling strategy
		(*e*) If applicable, describe any sensitivity analyses
**Results**		
Participants	13*	(a) Report numbers of individuals at each stage of study—eg numbers potentially eligible, examined for eligibility, confirmed eligible, included in the study, completing follow-up, and analysed
		(b) If applicable, give reasons for non-participation at each stage
		(c) If applicable, consider use of a flow diagram
Descriptive data	14*	(a) Give characteristics of study participants (eg demographic, clinical, social) and information on exposures and potential confounders
		(b) If applicable, Indicate number of participants with missing data for each variable of interest
		(c) *Cohort study*—If applicable, summarise follow-up time (eg, average and total amount)
Outcome data	15*	*Cohort study*—Report numbers of outcome events or summary measures over time
		Case-control study—Report numbers in each exposure category, or summary measures of exposure
		Cross-sectional study—Report numbers of outcome events or summary measures
Main results	16	(*a*) If applicable, give unadjusted estimates and, if applicable, confounder-adjusted estimates and their

		precision (eg, 95% confidence interval). Make clear which confounders were adjusted for and why they were included
		(*b*) If applicable, Report category boundaries when continuous variables were categorized
		(*c*) If applicable, If relevant, consider translating estimates of relative risk into absolute risk for a meaningful time period

Other analyses	17	Report other analyses done—eg analyses of subgroups and interactions, and sensitivity analyses
**Discussion**		
Key results	18	Summarise key results with reference to study objectives
Limitations	19	Discuss limitations of the study, taking into account sources of potential bias or imprecision. Discuss both direction and magnitude of any potential bias
Interpretation	20	Give a cautious overall interpretation of results considering objectives, limitations, multiplicity of analyses, results from similar studies, and other relevant evidence
Generalisability	21	Discuss the generalisability (external validity) of the study results
**Other information**		
Funding	22	Give the source of funding and the role of the funders for the present study and, if applicable, for the original study on
	23	which the present article is based
	24	Local relevance of the research question indicated/mentioned anywhere in the paper
		Ethics statement included
		Adherence to STROBE guidelines mentioned anywhere in the manuscript (*this will not be included in the overall denominator)

*Give information separately for cases and controls in case-control studies and, if applicable, for exposed and unexposed groups in cohort and cross-sectional studies.

For items 1a and 1b, a positive response will be scored as 0.5 each.

Items 12, 13, 14 and 16, have multiple components, each of which may (or may not be) applicable. The total applicable components will be considered in scoring. For example, item 12 has components 12a to 12e. If components 12a and 12b are applicable to a given study and both are reported, the score will be 1. If only 12a is reported and not 12b, this will be considered as a score of 0.5.

Some items (6a, 6b, 12d, 14c, 15) are specific for some study designs only (e.g. cohort or case control). Consequently, if an item was not applicable for the study design, it will be scored as `not applicable'. Please divide the number of adequately reported items by the total number of applicable items, which will give a proportion of adequately reported items.

Note: An Explanation and Elaboration article discusses each checklist item and gives methodological background and published examples of transparent reporting. The STROBE checklist is best used in

conjunction with this article (freely available on the web sites of PLoS Medicine at http://www.plosmedicine.org/, Annals of Internal Medicine at http://www.annals.org/, and Epidemiology at http://www.epidem.com/). Information on the STROBE Initiative is available at www.strobe-statement.org.

Publisher's Note: MDPI stays neutral with regard to jurisdictional claims in published maps and institutional affiliations.

©2020 World Health Organization; Licensee MDPI, Basel, Switzerland. This is an openaccess article distributed under the terms of the Creative Commons Attribution IGO License(http://creativecommons.org/licenses/by/3.0/igo/legalcode), which permits unrestricteduse, distribution, and reproduction in any medium, provided the original work is properlycited. In any reproduction of this article there should not be any suggestion that WHO orthis article endorse any specific organisation or products. The use of the WHO logo isnot permitted
